# Characterizing gene-gene interactions in a statistical epistasis network of twelve candidate genes for obesity

**DOI:** 10.1186/s13040-015-0077-x

**Published:** 2015-12-29

**Authors:** Rishika De, Ting Hu, Jason H. Moore, Diane Gilbert-Diamond

**Affiliations:** 1 Department of Genetics, Geisel School of Medicine at Dartmouth, Hanover, NH USA; 2 Department of Computer Science, Memorial University, St. John’s, NL Canada; 3 Institute for Biomedical Informatics, The Perelman School of Medicine, University of Pennsylvania, Philadelphia, PA USA; 4 Department of Epidemiology, Geisel School of Medicine at Dartmouth, Hanover, NH USA

**Keywords:** Dyadicity, Heterophilicity, Statistical epistasis networks, Epistasis, Gene-gene interaction

## Abstract

**Background:**

Recent findings have reemphasized the importance of *epistasis*, or gene-gene interactions, as a contributing factor to the unexplained heritability of obesity. Network-based methods such as statistical epistasis networks (SEN), present an intuitive framework to address the computational challenge of studying pairwise interactions between thousands of genetic variants. In this study, we aimed to analyze pairwise interactions that are associated with Body Mass Index (BMI) between SNPs from twelve genes robustly associated with obesity (*BDNF, ETV5, FAIM2, FTO, GNPDA2, KCTD15, MC4R, MTCH2, NEGR1, SEC16B, SH2B1,* and *TMEM18*).

**Methods:**

We used information gain measures to identify all SNP-SNP interactions among and between these genes that were related to obesity (BMI > 30 kg/m^2^) within the Framingham Heart Study Cohort; interactions exceeding a certain threshold were used to build an SEN. We also quantified whether interactions tend to occur more between SNPs from the same gene (*dyadicity*) or between SNPs from different genes (*heterophilicity*).

**Results:**

We identified a highly connected SEN of 709 SNPs and 1241 SNP-SNP interactions. Combining the SEN framework with dyadicity and heterophilicity analyses, we found 1 dyadic gene (*TMEM18, P-*value = 0.047) and 3 heterophilic genes (*KCTD15, P-*value = 0.045; *SH2B1, P-*value = 0.003; and *TMEM18, P-*value = 0.001). We also identified a lncRNA SNP (rs4358154) as a key node within the SEN using multiple network measures.

**Conclusion:**

This study presents an analytical framework to characterize the global landscape of genetic interactions from genome-wide arrays and also to discover nodes of potential biological significance within the identified network.

**Electronic supplementary material:**

The online version of this article (doi:10.1186/s13040-015-0077-x) contains supplementary material, which is available to authorized users.

## Background

By 2030, the obesity epidemic has the potential to affect 1.2 billion people worldwide [1]. Within the United States, a third of the adult population is categorized to be obese; this creates an estimated economic burden of $147 billion each year [2, 3]. Moreover, obesity has also been attributed to be a risk factor for conditions such as cardiovascular disease, type 2 diabetes, cancer and premature death [4]. Therefore, this issue has drawn the focus of many public health efforts in the U.S. These efforts have been especially important for combatting rising levels of childhood obesity [2].

In addition to the influence of environmental and lifestyle factors, obesity also has a strong genetic component. It has been shown to have heritability estimates ranging between 40 and 70 % [5, 6]. However, the genetic loci that have been found to be associated with Body Mass Index (BMI) so far, can explain only a portion of its variation [7]. *Epistasis* or gene-gene interactions are a possible contributing factor to this ‘*missing heritability*’ [8, 9].

Previously, genetic variants within *FTO* have been identified to exhibit the strongest association with obesity in humans [3, 10–12]. However, recent studies have found that these *FTO* variants are in fact associated with the expression levels of a nearby gene, *IRX3* [13]*.* Such findings have reemphasized the importance of gene-gene interactions in obesity. We aim to extend this work by studying interactions between twelve candidate obesity genes that have been consistently identified by multiple genome-wide association studies (GWAS) [7, 14–16]. Variants on these genes represent some of the strongest independent genetic associations that have been identified for BMI and account for ~1 % of the variance observed in BMI [17].

We employed previously established network science methodologies to construct a genetic interaction network and characterize epistatic interactions within this network [18, 19]. The use of networks provides an intuitive framework for studying and visualizing complex relationships between large numbers of biological entities [20]. A network is usually represented as a collection of vertices or nodes that are connected in pairs by edges. In addition to studying the properties of the nodes within this network, we also analyzed the distribution of certain node properties in relation to the underlying network structure. Park et al. have proposed the network metrics of *dyadicity* and *heterophilicity* in order to identify if interactions tend to occur more between nodes with similar characteristics [21]. We utilized these metrics in conjunction with a statistical epistasis network (SEN) to characterize gene-gene interactions associated with BMI within the Framingham Heart Study cohort.

## Methods

### Study cohort

**Fig. 1:**
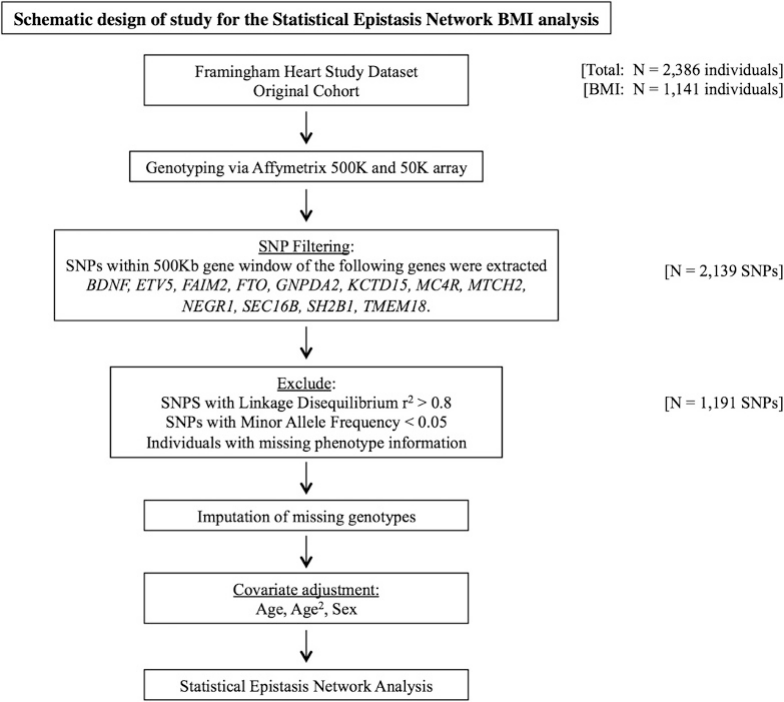
Schematic overview of analysis of Statistical Epistasis Network analysis of SNP-SNP interactions associated with BMI in the Framingham Heart Study data

The overall study design is illustrated in Fig. 1. We combined genotypic and phenotypic information for an initial population of 2386 individuals (1133 males, 1253 females) belonging to the original cohort of the NHLBI Framingham Heart Study. This study began in 1948 and was initially designed to identify common factors that contribute to cardiovascular disease [22].

### Phenotype information

The original cohort included participants between the ages of 29 to 61 years, who returned every two years for a physical examination and lifestyle interviews. Phenotype information was used from the first physical exam performed. Weight was measured to the nearest pound. Height was measured to the nearest inch.

Body Mass Index (BMI) was calculated according to the following formula [4]:

$$ BMI = \frac{Weight\ \left( in\  kg\right)}{Heigh{t}^2\ \left( in\ m\right)} $$

Individuals were categorized into obese (BMI > 30 kg/m^2^) and non-obese (BMI <30 kg/m^2^). Age and sex were obtained from the exam questionnaires.

### Genotype information

We focused our analysis on SNPs belonging to the following twelve genes – *BDNF, ETV5, FAIM2, FTO, GNPDA2, KCTD15, MC4R, MTCH2, NEGR1, SEC16B, SH2B1,* and *TMEM18* [7, 14–16]. SNPs belonging to these genes were extracted from genotype files using the PLINK --extract and --range commands [23]. SNPs were considered to fall in a gene if they were within a 500Kb window around the gene. Chromosomal locations used for defining each genomic region are listed in Additional file 1: Table S1. This was done to ensure the inclusion of potential regulatory genetic variants in the region as well.

Study participants were genotyped using the Affymetrix 500 K mapping array and the Affymetrix 50 K supplemental array. SNPs with a minor allele frequency < 0.05 were excluded. SNPs were further tested for linkage disequilibrium (LD) – a SNP was removed from each pair of SNPs that showed an LD (r^2^) value > 0.8. Additionally, missing genotypes were imputed using the most frequent genotype for a given marker across all individuals. This resulted in a final dataset of 1191 SNPs for 1141 individuals.

### Statistical epistasis network construction

We utilized a previously developed informatics framework, Statistical Epistasis Networks (SEN), that is able to characterize the global structure of interactions between genetic variants from GWA studies [18]. The SEN method only considers purely epistatic interactions, i.e., it measures the effect of the interaction outside of the individual main effects of the interacting SNPs. This is in contrast to the traditional linear regression method of studying interactions, which is unable to disentangle main effects and purely epistatic interactions.

Dichotomized BMI values were adjusted for age, age^2^ and sex using a generalized linear model. Individuals with deviance residual BMI values > 0 were classified as ‘cases’; otherwise they were ‘controls’. This classification was used as the phenotype outcome in the network analysis. Additionally, there was a 100 % concordance in classification of individuals before and after covariate adjustment.

As an initial step, all pairwise epistatic interactions between SNPs were evaluated using ‘*information gain*’ – a metric used in Information Theory.

Before explaining *information gain*, we first introduce the concept of *entropy*, which is a measure of the uncertainty of a random variable [24, 25]. It can be explained as the average amount of information required to describe a random variable. Hence, *entropy* is at its maximum when all possible outcomes of a process can occur with equal probability; as predictability of an outcome increases *entropy* decreases.

For a discrete variable X with alphabet *X* and probability mass function *p(x)*, the entropy *H(X)* is calculated as follows [24–26]:

$$ H(X) = -{\displaystyle \sum_{x\in X}}p(x) \log p(x) $$

Furthermore, the dependency between two random variables can be understood using *mutual information* [24, 26]. In genetic association studies, *mutual information* is useful for quantifying how much of a phenotype can be explained by genetic variants. For a SNP *A* and phenotype *C, mutual information* is calculated as follows:

$$ I\left(A;C\right) = H(C)-H\left(C\Big|A\right) $$

where *H(C)* is the measure of the *entropy* or the uncertainty of *C*, and *H(C|A)* is the measure of *conditional entropy* of *C* given the knowledge of SNP *A*. Hence, *mutual information* describes the reduction in the uncertainty of the phenotype *C* due to the knowledge of genotype *A*. Intuitively, *mutual information* can be used as a measure of the independent or main effect of SNP *A* on phenotype *C*.

The concept of *mutual information* can also be used to study the interaction effect between a pair of SNPs *A* and *B*. It explains how much of the phenotype *C* can be understood when both genotypes are combined.

Thus, by subtracting the individual associations of SNPS *A* and *B* on *C* – *I(A;C)* and *I(B;C)* – from the total association of both SNPs, *I(A,B;C)*, we can calculate the *information gain* or the gain in mutual information using the formula below [27]:

$$ IG\left(A;B;C\right) = I\left(A,B;C\right) - I\left(A;C\right) - I\left(B;C\right) $$

The *information gain* metric serves as a measure of the epistatic interaction, or synergy, between the two SNPs on explaining the phenotypic outcome *C*.

Within the SEN, each vertex or node corresponds to a certain SNP. The edge or connection between two nodes represents the interaction between the two SNPs. The weight of a node, or the strength of the main effect of that SNP, is represented as the size of the node. Larger node sizes correspond to stronger main effects. Lastly, the weight of an edge represents the strength of the epistatic interaction between two SNPs. Thicker edges correspond to stronger interactions [18].

The SEN was built using pairwise interactions that are stronger than a theoretically derived threshold. By gradually adjusting the edge-weight threshold, a series of networks were constructed. By inspecting the network topology, we identified the most significant threshold that resulted in a network that was the most different from what was expected by chance. In this study, we used a *percolation threshold*, i.e., the first time more than half the nodes of the network were connected in the largest connected component. It can be thought of as an inflection point such that after this threshold the connectivity of the network changes rapidly [25].

We also generated 1000 permuted datasets by randomly shuffling the phenotype status to reflect the null hypothesis that there is no association between the genotypes and the phenotype. For each permuted dataset, a series of networks were constructed with the same thresholds used to build the real-data networks. These permuted-data networks were used to build a null distribution to assess the statistical significance of various properties of the real-data network.

### Network analysis

*Dyadicity* and *heterophilicity* are two normalized network metrics that are used to measure the correlation between node properties and the underlying network structure. Park and Barabási proposed these measures as part of an approach to assess whether vertices with similar properties tend to be connected with each other in a network [21].

**Fig. 2:**
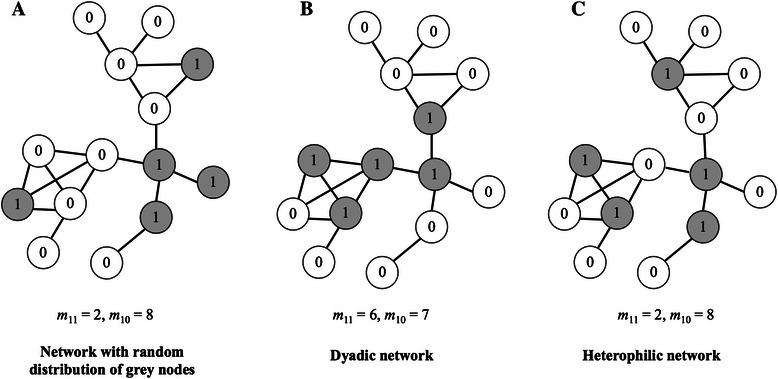
Examples of dyadic and heterophilic distributions of node properties in a network. Nodes can be classified as either 0 or 1 (white or grey respectively). **a** shows a network on which 5 grey nodes are distributed randomly. **b** For a certain number of nodes, if edges occur more between similar nodes (i.e., 1–1 edges) than expected at random, the network is dyadic. **c** If edges occur more between dissimilar nodes (i.e., 1–0 edges) than at random, then the network is heterophilic

We first explored whether interactions tend to occur more between SNPs from different genes or from the same gene. In the context of this SEN, a node can be characterized by a property that takes the value of 0 or 1 (Fig. 2) [28]. In our analysis, this signifies that a certain SNP belongs to a certain gene (1) or not (0). There are three possible types of *dyads*, i.e., an edge and its two nodes – i) an edge and two vertices that both have the value 1, ii) an edge and two vertices with values 1 and 0, and iii) an edge and two vertices with the value 0 [21, 28]. The expected number of (1-1) and (1-0) dyads in the network are denoted as ${\overline{m}}_{11}$ and ${\overline{m}}_{10}$ respectively [21, 28]. Thus *dyadicity* (*D*) and *heterophilicity* (*H*) are calculated as follows:

$$ \begin{array}{l}D = \frac{m_{11}}{{\overline{m}}_{11}}\\ {}H = \frac{m_{10}}{{\overline{m}}_{10}}\end{array} $$

where *m*_11_ and *m*_10_ are the observed number of dyads in the network [21, 28]. This ensures that the measures of *D* and *H* are normalized and account for any variability due to the differences in the number of nodes assigned with the values of 0 or 1. A statistically significant deviation of *D* and *H* from 1 symbolizes a non-random distribution of the property in the network [21, 28]. Hence, a value of *D* > 1 signifies a dyadic network; a network where nodes with similar properties tend to connect with each more than expected (Fig. 2b). Similarly, a value of *H* > 1 signifies a heterophilic network (Fig. 2c). In such a network, nodes with a certain property tend to be more connected with nodes without that property, than expected at random.

In our analyses, we used these two metrics *D* and *H*, to assess whether interactions tend to occur more between SNPs from different genes or from the same gene. Statistical significance of the observed values of *D* and *H* was assessed using permutation testing as well. A 1000-fold permutation test was performed where the network structure and the total number of nodes with the value 1 were fixed. Next, the node value assignments of 0 and 1 were reassigned randomly, and *D* and *H* values were calculated. These values were used to build the permutation distribution used to assess the statistical significance of the *D* and *H* values of our real-data network.

In addition to characterizing the global network, a few other measures of node properties were also utilized to identify key nodes within the SEN, such as – *degree*, *betweenness centrality* and *closeness centrality*. These measures highlight the fact that not all nodes within a network are considered to be of equal importance. The *degree* of a node refers to the number of edges connecting to it [29]. Nodes with a high degree are often referred to as ‘*hubs’* [30]. *Betweenness centrality* is a measure of the number of shortest paths that go through a node. Nodes exhibiting high betweenness centrality are often viewed as ‘bottlenecks’ of information flow, since they connect two disparate portions of a network [30]. *Closeness centrality* is calculated as the reciprocal of the sum of the total distance to all other vertices in the network [31].

### Integrated Multi-Species Prediction (IMP) web server

We also used the Integrated Multi-Species Prediction (IMP) web server to query genes represented by the SNPs within the most significant pairwise interaction [32]. IMP serves as a repository that combines biological evidence from multiple sources such as gene expression studies, IntAct, MINT, MIPS, and BioGRID databases. The software then mines such empirical data to provide a probability score that two genes are involved in a functional and biological relationship.

## Results

### Gene categorization

SNPs included in the study were chosen from twelve candidate obesity genes. Additional file 1: Table S1 shows the number of SNPs that were included from each gene. The known biological roles of each of these genes are also described in detail (Additional file 1: Table S1).

### Statistical epistasis network

**Fig. 3:**
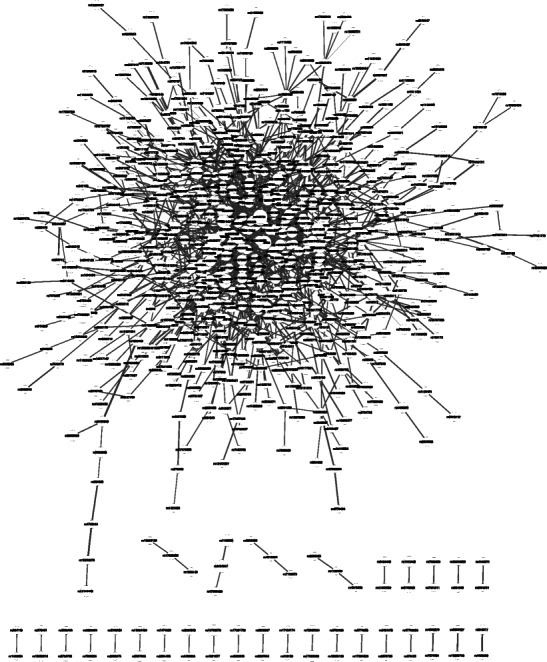
Statistical Epistasis Network of SNPs from 12 candidate obesity genes associated with BMI. Each node represents a SNP, and each edge connecting two nodes represents an interaction. Shown here is the SEN at a percolation threshold of 0.023379, with 771 nodes and 1241 edges. The largest connected component is comprised of 709 nodes. This network was rendered using Cytoscape

At a percolation threshold of 0.023379, we identified a highly connected SEN comprised of 771 SNPs as nodes and 1241 edges (Fig. 3). The number of nodes included in our SEN was statistically significant with a *P*-value = 0.036. The largest connected component consisted of 709 SNPs and has a statistically significant size compared to all 1000 permuted-data networks at the same threshold with a *P*-value = 0.046.

### Measures of main effect and interaction strength

**Fig. 4:**
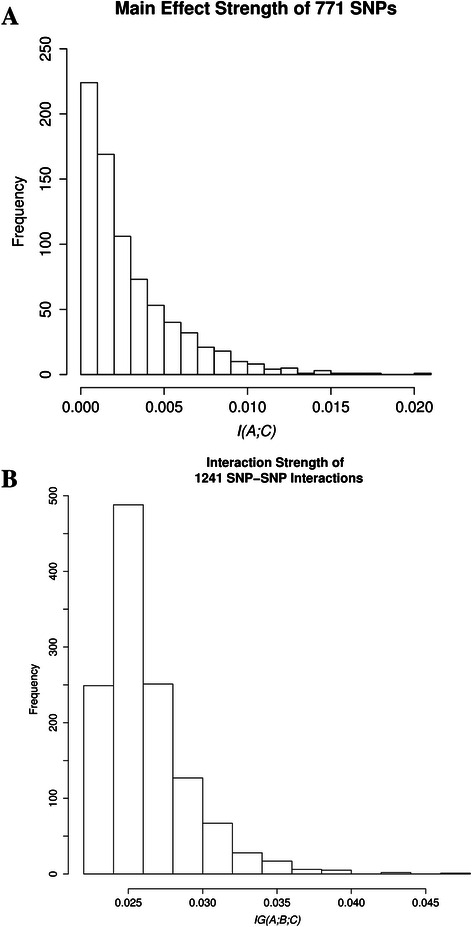
Frequency distributions of mutual information and information gain in the Statistical Epistasis Network. Shown here are the distributions of **a** the main effect strengths of 771 SNPs and **b** the interaction strengths of 1241 SNP-SNP interactions associated with BMI within the network

Figure 4a shows the frequency distribution of the main effect strengths or mutual information values of all individual SNPs associated with BMI, within the network at the percolation threshold. Within this network, we identified 58 SNPs with a statistically significant main effect (*P*-value < 0.05) (Additional file 2: Table S2). The 5 strongest main effects are shown in Table 1.

**Table 1:** Mutual information values for five SNPs with the strongest main effects associated with BMI within the Statistical Epistasis Network at the percolation threshold

SNP	Gene	Mutual Information	Permuted *P*-value
rs17066891	MC4R	0.020138781	<0.001
rs9940128	FTO	0.01695201	0.001
rs1866510	GNPDA2	0.017895503	0.002
rs9949577	MC4R	0.014867522	0.002
rs12696555	ETV5	0.013757477	0.004

The corresponding gene for each SNP is also shown. Main effect strengths are measured using mutual information [I(A;C)]. *P*-values were calculated from a 1000 permutations

The frequency distribution of interaction strengths of all 1241 pairwise SNP-SNP interactions associated with BMI, within the network at the percolation threshold is shown in Fig. 4b. All the interactions were highly significant with a *P*-value < 0.001 (Additional file 3: Table S3). The five strongest interactions are shown in Table 2.

**Table 2:** Shown are the five SNP-SNP interactions with the highest information gain values for BMI within the Statistical Epistasis Network at the percolation threshold

Interaction	SNP1	Gene1	SNP2	Gene2	Information Gain	Permuted *P*-value
rs2867133,rs9878325	rs2867133	TMEM18	rs9878325	ETV5	0.0473789	<0.001
rs7110708,rs8105874	rs7110708	BDNF	rs8105874	KCTD15	0.043324175	<0.001
rs17360705,rs1673518	rs17360705	SEC16B	rs1673518	MC4R	0.042418682	<0.001
rs10798574,rs2245826	rs10798574	SEC16B	rs2245826	BDNF	0.03917757	<0.001
rs8179316,rs1316803	rs8179316	NEGR1	rs1316803	TMEM18	0.038534544	<0.001

The corresponding gene for each SNP is also shown. SNP-SNP interactions are measured using information gain [IG(A;B;C)]. *P*-values were calculated from a 1000 permutations

### Dyadicity and heterophilicity of gene categories

Table 3 shows the dyadicity and heterophilicity values for each of the twelve candidate obesity genes. *TMEM18* was the only gene that showed significant dyadicity (*P*-value = 0.04). Three genes showed significant heterophilicity: *TMEM18* (*P*-value <0.001), *SH2B1* (*P*-value = 0.001) and *KCTD15* (*P*-value = 0.038). Additional file 4: Figure S1; Additional file 5: Figure S2; Additional file 6: Figure S3 and Additional file 7: Figure S4 show the null distributions used to assess the statistical significance of the dyadicity and heterophilicity values of *TMEM18*, *SH2B1*, and *KCTD15* from 1000 permuted networks.

**Table 3:** Results from dyadicity and heterophilicity analysis of the statistical epistasis network. Shown are the dyadicity and heterophilicity values for each of the twelve candidate obesity genes

Gene	*n*_1_	*m*_11 expected_	*m*_10 expected_	*m*_11 observed_	*m*_10 observed_	*D*	*H*	*P*-value_*D*_	*P*-value_*H*_
FTO	324	28.399	319.157	21	301	0.739	0.943	0.85	0.727
MC4R	187	9.439	198.109	16	219	1.695	1.105	0.074	0.248
KCTD15	178	8.550	189.444	5	243	0.585	1.283	0.855	**0.045**
TMEM18	222	13.314	230.971	23	334	1.728	1.446	**0.047**	**0.001**
NEGR1	295	23.535	295.234	12	249	0.510	0.843	0.978	0.94
SH2B1	32	0.269	36.593	1	87	3.715	2.378	0.232	**0.003**
FAIM2	106	3.020	116.957	1	103	0.331	0.881	0.91	0.699
SEC16B	165	7.343	176.772	2	197	0.272	1.114	0.979	0.218
ETV5	168	7.613	179.713	3	173	0.394	0.963	0.952	0.596
BDNF	167	7.523	178.734	1	167	0.133	0.934	0.997	0.643
MTCH2	109	3.195	120.090	1	110	0.313	0.916	0.908	0.664
GNPDA2	186	9.338	197.151	1	125	0.107	0.634	1	0.997

*P*-values were calculated from 1000 permutations. *P*-values <0.05 are highlighted in bold

### Measures of node properties – degree, betweenness centrality and closeness centrality

Values for degree, betweenness centrality and closeness centrality were calculated for all SNPs within the largest connected component of the network (Additional file 8: Table S4). The corresponding frequency distribution of these three node properties is presented in Additional file 9: Figure S5; Additional file 10: Figure S6 and Additional file 11: Figure S7. Table 4 shows the SNPs with the 5 highest values for each of these measures. rs4358154 in *TMEM18* had the highest value for all three measures.

**Table 4:** Shown here are the five SNPs with the highest degree, betweenness centrality and closeness centrality scores respectively, amongst all SNPs in the largest connected component of the Statistical Epistasis Network

SNP	Gene	Degree
rs4358154	TMEM18	22
rs285690	KCTD15	18
rs3817334	MTCH2	17
rs529579	KCTD15	17
rs4650977	SEC16B	17
SNP	Gene	Betweenness Centrality
rs4358154	TMEM18	0.08366177
rs4650977	SEC16B	0.06191228
rs2278260	KCTD15	0.05311856
rs529579	KCTD15	0.05173649
rs10742817	MTCH2	0.05150078
SNP	Gene	Closeness Centrality
rs4358154	TMEM18	0.29611041
rs3817334	MTCH2	0.29598662
rs2278260	KCTD15	0.29377593
rs17066403	MC4R	0.29304636
rs529579	KCTD15	0.29256198

The corresponding gene for each SNP is also shown

## Discussion

The need for embracing the complexity of data from genome-wide genotyping arrays, also presents a bioinformatics challenge. Trying to study interactions between thousands of genetic variants can be computationally demanding. However, this is important for truly elucidating the disease mechanisms of complex disorders. The use of network science and information theory provides an intuitive framework for representing the inter-connectedness between biological entities and assessing the global structure of these interactions. It also enables researchers to identify key network nodes by studying the interplay between global network properties and node properties. Studying gene-gene interactions has been especially important in the context of obesity, as shown in recent studies.

In this study, we constructed an SEN of SNPs from twelve candidate obesity genes. SNPs belonging to these genes were filtered from the Framingham Heart Study dataset. Initially, all pairwise SNP-SNP interactions associated with BMI were calculated, using the ‘*information gain’* measure. Next, SNP-SNP interactions exceeding a certain threshold were used to construct the network. The corresponding gene for each SNP was also overlaid onto this network. This was used in combination with the network measures of *dyadicity* and *heterophilicity* to investigate the nature of interactions within the SEN. We aimed to understand if interactions tend to occur more between different genes or within the same gene.

We identified a highly connected SEN that had a largest connected component consisting of nearly 90 % of the total number of SNPS (709 out of 771 SNPs) in the network. This reflects the complex interconnectedness that may be playing into the disease mechanism of obesity. rs17066891 in *MC4R* was identified as having the strongest main effect within this network (Table 1). To the best of our knowledge, this SNP has not been implicated in obesity previously. The SNP rs9940128 in *FTO* was identified as having the second strongest main effect in the network (Table 1). This SNP has been previously identified to be associated with BMI with a genome-wide significance, in adolescents and young adults [33].

The information gain measure is mathematically designed for identifying synergistic interactions that help explain a phenotype, beyond what is learned about it through the independent effects of SNPs. The SNP-SNP interaction with most information gained about BMI is between rs2867133 in *TMEM18* and rs9878325 in *ETV5* (Table 2). *TMEM18* has been found to be widely expressed in the brain, including the hypothalamus – the region responsible for controlling the feeling of satiety [34]. This finding corresponds with the previously established role of the central nervous system (CNS) in obesity [35]. *ETV5* encodes for a transcription factor belonging to the ETS family [36].

**Fig. 5:**
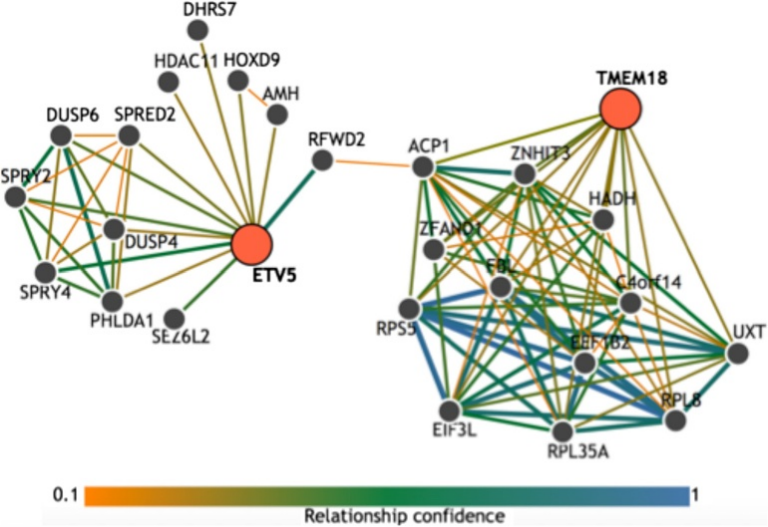
Functional relationship network generated from Integrated Multi-Species Prediction (IMP) reflecting the BMI associated SNP-SNP interaction between rs2867133 in *TMEM18* and rs9878325 in *ETV5*. SNPs were mapped to their respective genes and used to query IMP. Nodes in the network represent genes. Orange nodes are the genes that were queried. Edges between nodes represent a functional relationship between two genes. The color of the edge signifies the strength of the relationship confidence

Using IMP, we identified a functional relationship connecting *TMEM18* and *ETV5* (Fig. 5) [32]. *ETV5* is known to physically interact with the E3 ubiquitin protein ligase encoded by *RFWD2* [32]. This ubiquitin ligase interacts with ACP1, a phosphatase [32]. There was also some support for the interaction between ACP1 and TMEM18 in the network. TMEM18 is known to have conserved phosphorylation sites [36]. Hence, the interaction between *ETV5* and *TMEM18* could be highlighting a regulatory relationship. ACP1 may be involved in the post-translational modification of TMEM18. Moreover, the possible degradation of ACP1 due to ubiquitination could add an additional layer of regulation.

We also performed dyadicity and heterophilicity analyses to characterize the gene-gene interactions within the SEN. We identified three genes with significant heterophilicity (*SH2B1, KCTD15* and *TMEM18*), and one gene with significant dyadicity (*TMEM18*). Heterophilic genes were involved in more SNP-SNP interactions with other genes than expected at random. *SH2B1* encodes for a cytoplasmic adaptor protein and has been implicated in leptin signaling [37]. The significant heterophilicity of this gene may be due to the fact that adaptor proteins contribute to the cross-talk between various signaling cascades by bringing together larger protein complexes [38]. Moreover, the obesity observed in *Sh2b1*-null mice was reversed by the targeted expression of *Sh2b1* in neurons [37]. This was important for highlighting the role of the CNS in the development of obesity, since *SH2B1* is expressed both in the CNS and peripheral tissues [35, 37]. The other two heterophilic genes *TMEM18* and *KCTD15* have unknown functions but are known to be highly expressed in the hypothalamus and brain [35]. Ultimately, these genes reemphasize the brain’s role in the development of obesity. Their significant heterophilicity may be a reflection of their biologically central role in regulating the actions of various cells and organs from within the brain.

*TMEM18* also showed marginally significant dyadicity (*P*-value = 0.04). Dyadic genes were part of more intra-genic interactions than expected at random. We identified 23 intra-genic interactions between SNPs in *TMEM18*, to be associated with BMI within the SEN (Additional file 3: Table S3). Although the biological effect of such interactions within *TMEM18* is unknown, these interactions may influence the gene’s function and obesity through regulatory and epigenetic mechanisms.

We also utilized network measures such as degree, betweenness centrality and closeness centrality to identify nodes within the SEN that may be of potential biological relevance. In the case of biological networks, certain nodes may play a more important role in the proper functioning of a cell [39] or may serve as better targets for intervention [40]. Researchers have found that hubs within a protein interaction network are encoded by essential genes in model organisms [39]. Moreover, in similar networks, proteins that are also bottlenecks, have been found to be of high biological significance and are often encoded by essential genes as well [41]. The closeness centrality measure has been utilized for identifying central nodes in various types of networks such as metabolic networks [42].

**Fig. 6:**
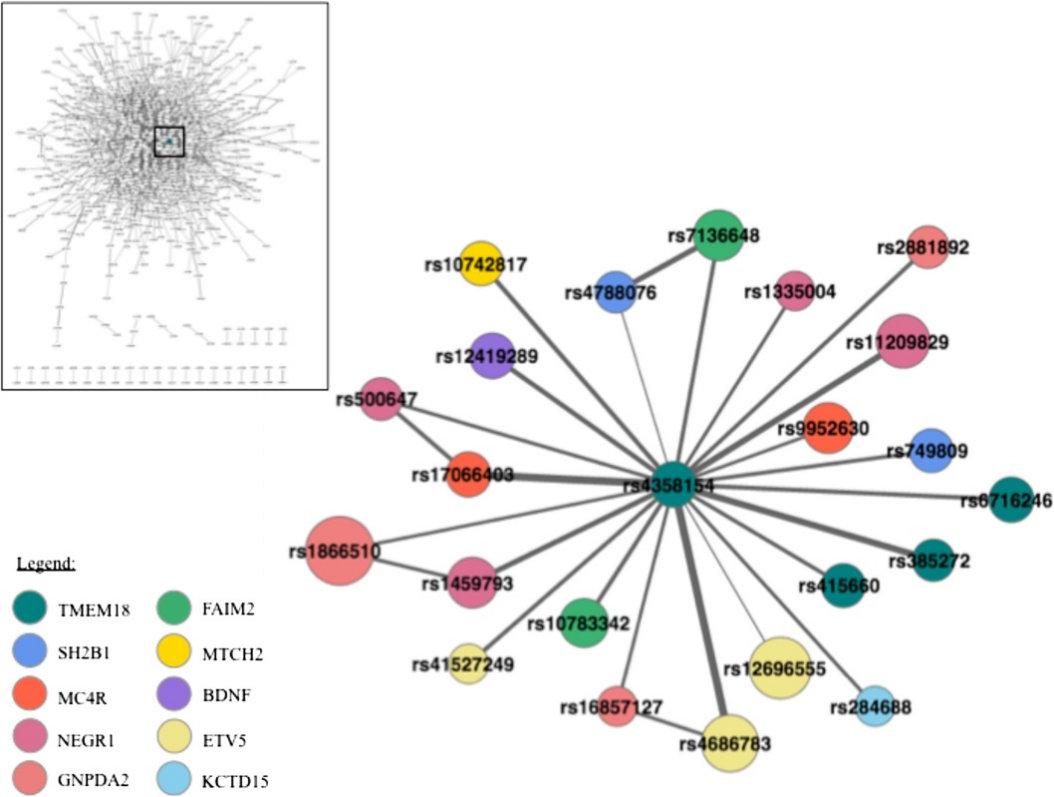
Focused view on the node (rs4358154 in *TMEM18)* showing the highest degree, betweenness centrality and closeness centrality within the Statistical Epistasis Network. Shown are all the direct neighboring nodes of rs4358154. Nodes are color coded based on their gene category. Node size represents the strength of the main effect of a SNP. The thickness of an edge represents the strength of the interaction between two SNPs

In our analyses, we identified a SNP (rs4358154) in *TMEM18* that had the highest score for all three measures described above (Table 3, Fig. 6). This not only highlights the potentially significant role of this SNP in the context of obesity, but may also represent the highly significant heterophilicity of *TMEM18* within the SEN. The SNP is of unknown function, but it is known to be located on *LINC01115,* a long intergenic non-protein coding RNA (lncRNA), located approximately 102 kb downstream of *TMEM18* [43]. Unfortunately, not much is known regarding the function of *LINC01115* as well. However, using the NONCODEv4 database, we found that this lncRNA shows most expression within the brain [44]. The regulatory role of lncRNAs has been investigated in various contexts including adipogenesis. Researchers have identified lncRNAs as a potential additional layer of regulation involved in the development of mature adipocytes or fat cells [45].

Understanding the regulatory mechanisms involved in the development of fat cells is of special importance for the advancement of future anti-obesity treatments. Humans have two types of fat cells – white and brown. Accumulation of white fat cells causes obesity since they store excess energy as fat or lipid droplets [46]. However, brown fat cells that are more abundant in infants use lipids as a fuel to maintain a warm body temperature [46]. Hence, researchers are interested in exploring the role of regulators such as lncRNAs in the development of each type of fat cell and learning how such processes may be manipulated.

## Conclusion

Exhaustively studying all pairwise interactions between SNPs from a genome-wide array can present a computationally challenging problem. In this study we used a network-based approach to investigate all pairwise interactions between SNPs in twelve candidate obesity genes within the Framingham Heart Study dataset. The use of this methodology enabled us to capture the landscape of interactions between genes known to be associated with BMI and to better understand which interactions are predictive of BMI. Furthermore, we were able to characterize these interactions, emphasize new roles of these genes and highlight the involvement of regulatory frameworks in the development of obesity.

## Additional files

Additional file 1: Table S1. Gene categorization. Shown here are the chromosomal locations (NCBI build 36) used to assign a SNP to a gene. Chromosomal location refers to the gene’s boundaries +/− a 500 kb window around it. The number of SNPs that were categorized to each of the 12 candidate obesity genes are also shown. Known biological roles of genes were found using GeneCards database (www.genecards.org, Accessed March 31, 2015) or from the literature sources cited. (XLSX 39 kb) Additional file 2: Table S2. Mutual information values for 58 SNPs within the network at the percolation threshold. Shown here are the statistically significant mutual information values (*p*-value < 0.05) of 58 SNPs within the network at the percolation threshold. *P*-values were calculated from a 1000 permutations. (XLSX 48 kb) Additional file 3: Table S3. Information gain values for 1241 edges within the network at the percolation threshold. Shown here are the statistically significant information gain values (*p*-value < 0.05) of 1241 edges within the network at the percolation threshold. *P*-values were calculated from a 1000 permutations. Also shown are the corresponding genes for each SNP. (XLSX 139 kb) Additional file 4: Figure S1. Null distribution of heterophilicity values of *KCTD15* from 1000 permuted networks. Null distribution of heterophilicity values of *KCTD15* from 1000 permuted networks. The red line indicates the observed heterophilicity value of *KCTD15* within the real data network. (PDF 12 kb) Additional file 5: Figure S2. Null distribution of heterophilicity values of *SH2B1* from 1000 permuted networks. Null distribution of heterophilicity values of *SH2B1* from 1000 permuted networks. The red line indicates the observed heterophilicity value of *SH2B1* within the real data network. (PDF 12 kb) Additional file 6: Figure S3. Null distribution of heterophilicity values of *TMEM18* from 1000 permuted networks. Null distribution of heterophilicity values of *TMEM18* from 1000 permuted networks. The red line indicates the observed heterophilicity value of *TMEM18* within the real data network. (PDF 12 kb) Additional file 7: Figure S4. Null distribution of dyadicity values of *TMEM18* from 1000 permuted networks. Null distribution of dyadicity values of *TMEM18* from 1000 permuted networks. The red line indicates the observed heterophilicity value of *TMEM18* within the real data network. (PDF 12 kb) Additional file 8: Table S4. Degree, betweenness centrality and closeness centrality values of 709 SNPs within the largest component of the network at the percolation threshold. Shown here are the values for degree, betweenness centrality and closeness centrality of 709 SNPs within the largest component of the Statistical Epistasis Network at the percolation threshold. (XLSX 81 kb) Additional file 9: Figure S5. Frequency distribution of node degree values within the SEN. Frequency distribution of node degree of 709 SNPs within the giant connected component of the SEN. The green line indicates the top 5 % of node degree values. (PDF 18 kb) Additional file 10: Figure S6. Frequency distribution of node betweenness centrality values within the SEN. Frequency distribution of node betweenness centrality measures of 709 SNPs within the giant connected component of the SEN. The green line indicates the top 5 % of betweenness centrality values. (PDF 20 kb) Additional file 11: Figure S7. Frequency distribution of node closeness centrality values within the SEN. Frequency distribution of node closeness centrality measures of 709 SNPs within the giant connected component of the SEN. The green line indicates the top 5 % of closeness centrality values. (PDF 21 kb)

## Abbreviations

BMI: Body mass index
GWAS: Genome wide association study
SEN: Statistical epistasis network
SNP: Single nucleotide polymorphism
LD: Linkage disequilibrium
