# Supplementary figures and images for: Characterizing gene-gene interactions in a statistical epistasis network of twelve candidate genes for obesity
Source: BioData Min. 2015 Dec 29;8:45. doi: 10.1186/s13040-015-0077-x (PMC4693412; doi:10.1186/s13040-015-0077-x)

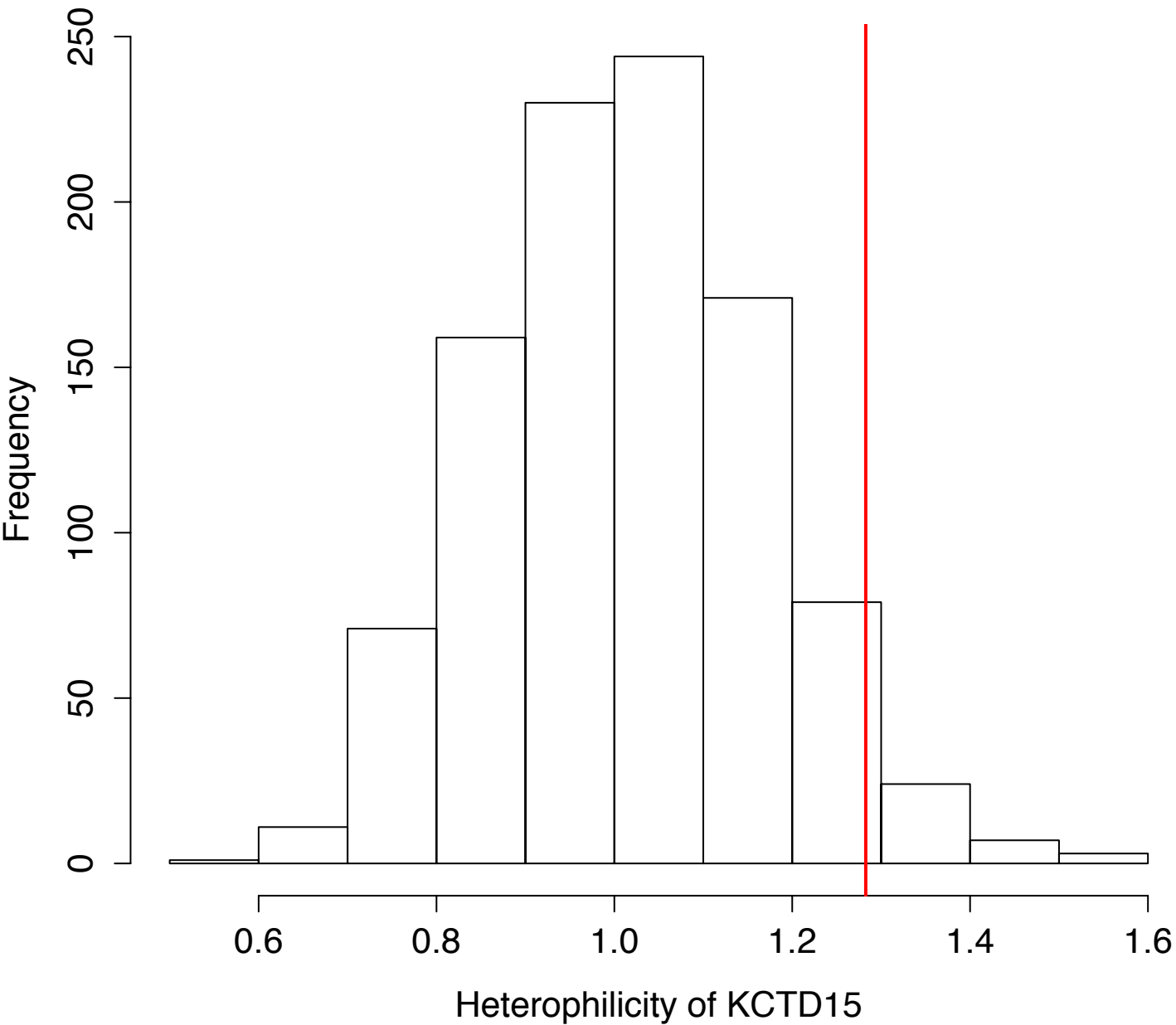

Supplement: Additional file 4: Figure S1. — Null distribution of heterophilicity values of KCTD15 from 1000 permuted networks. Null distribution of heterophilicity values of KCTD15 from 1000 permuted networks. The red line indicates the observed heterophilicity value of KCTD15 within the real data network. (PDF 12 kb) [file 13040_2015_77_MOESM4_ESM.pdf]

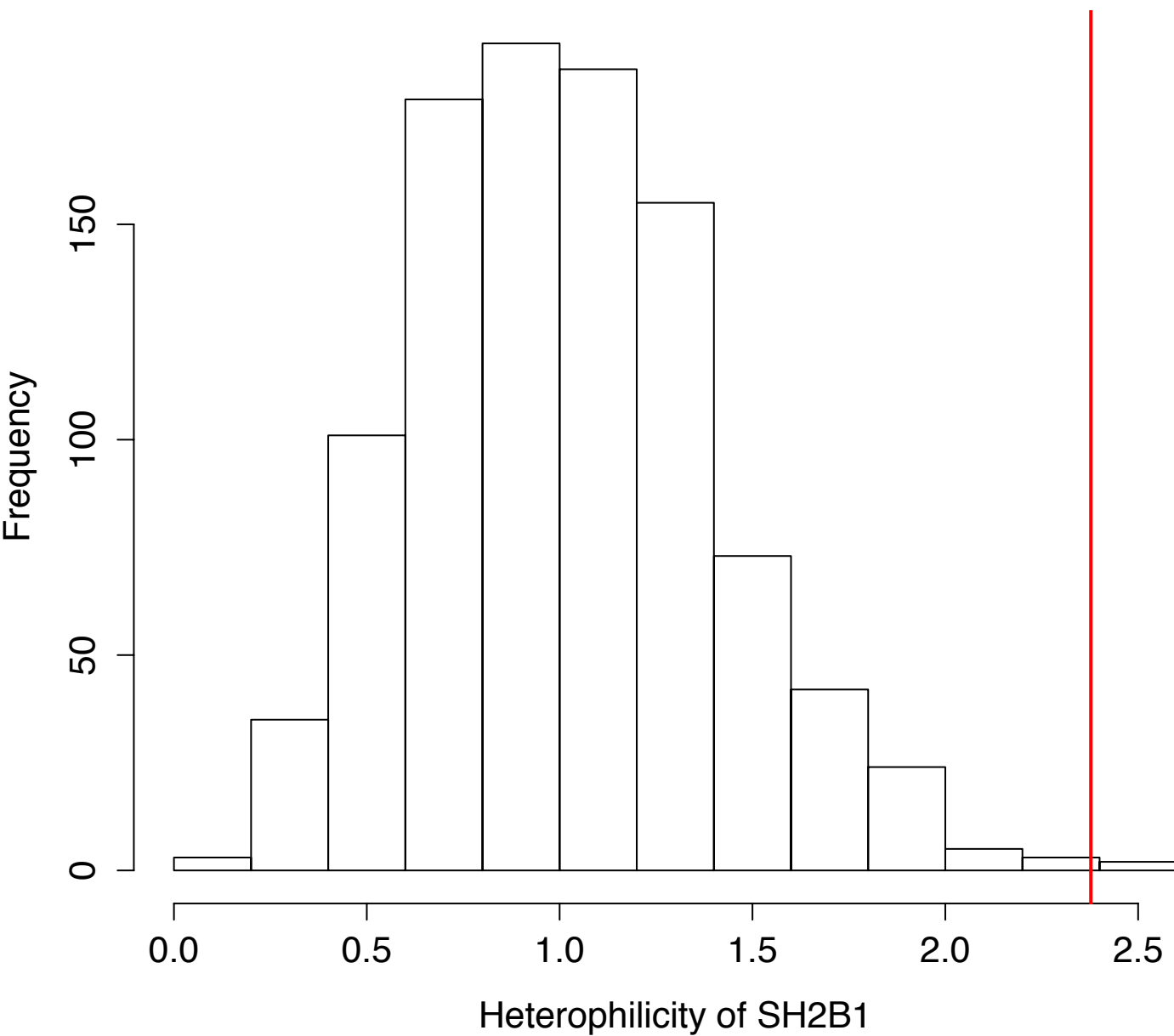

Supplement: Additional file 5: Figure S2. — Null distribution of heterophilicity values of SH2B1 from 1000 permuted networks. Null distribution of heterophilicity values of SH2B1 from 1000 permuted networks. The red line indicates the observed heterophilicity value of SH2B1 within the real data network. (PDF 12 kb) [file 13040_2015_77_MOESM5_ESM.pdf]

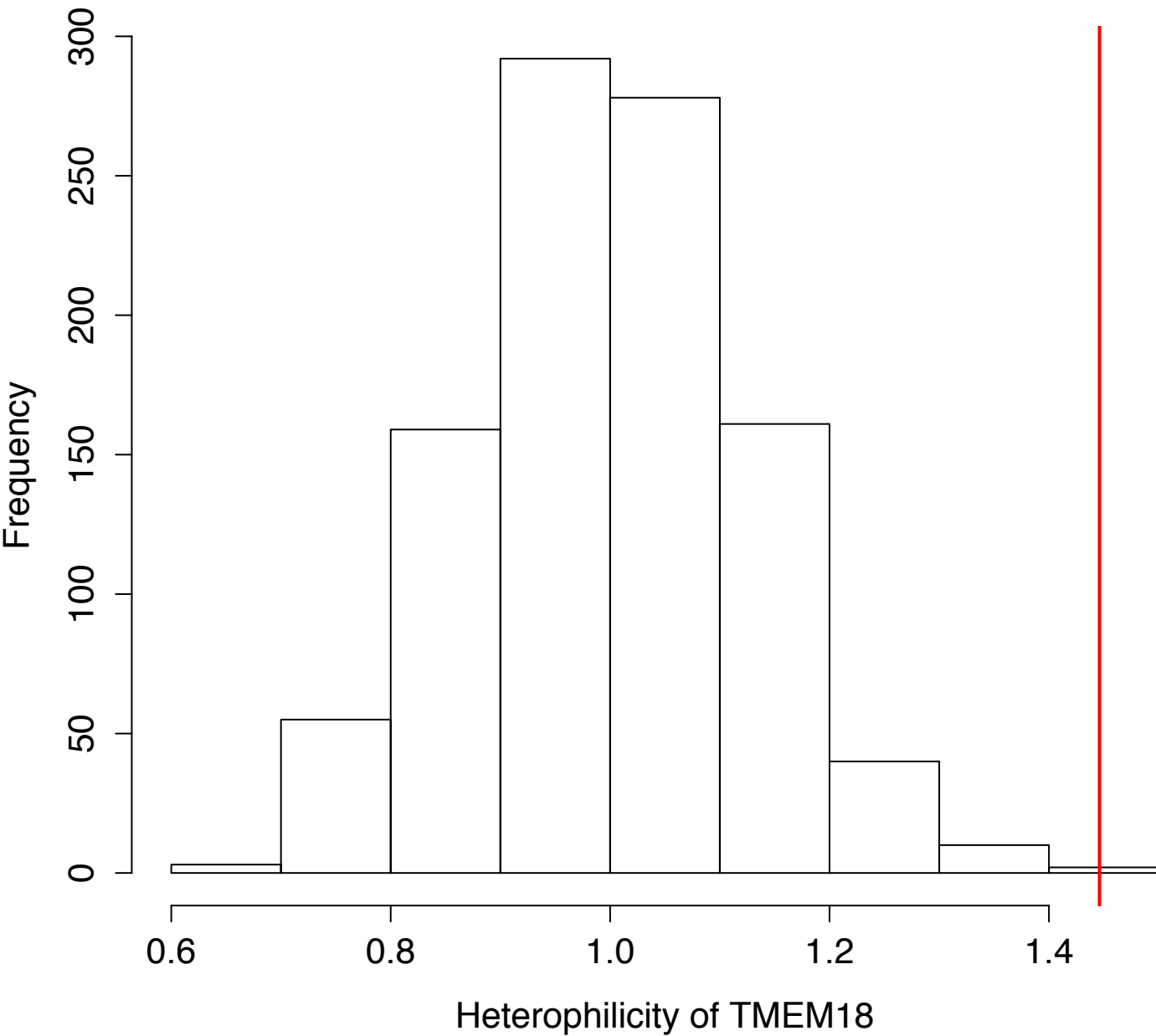

Supplement: Additional file 6: Figure S3. — Null distribution of heterophilicity values of TMEM18 from 1000 permuted networks. Null distribution of heterophilicity values of TMEM18 from 1000 permuted networks. The red line indicates the observed heterophilicity value of TMEM18 within the real data network. (PDF 12 kb) [file 13040_2015_77_MOESM6_ESM.pdf]

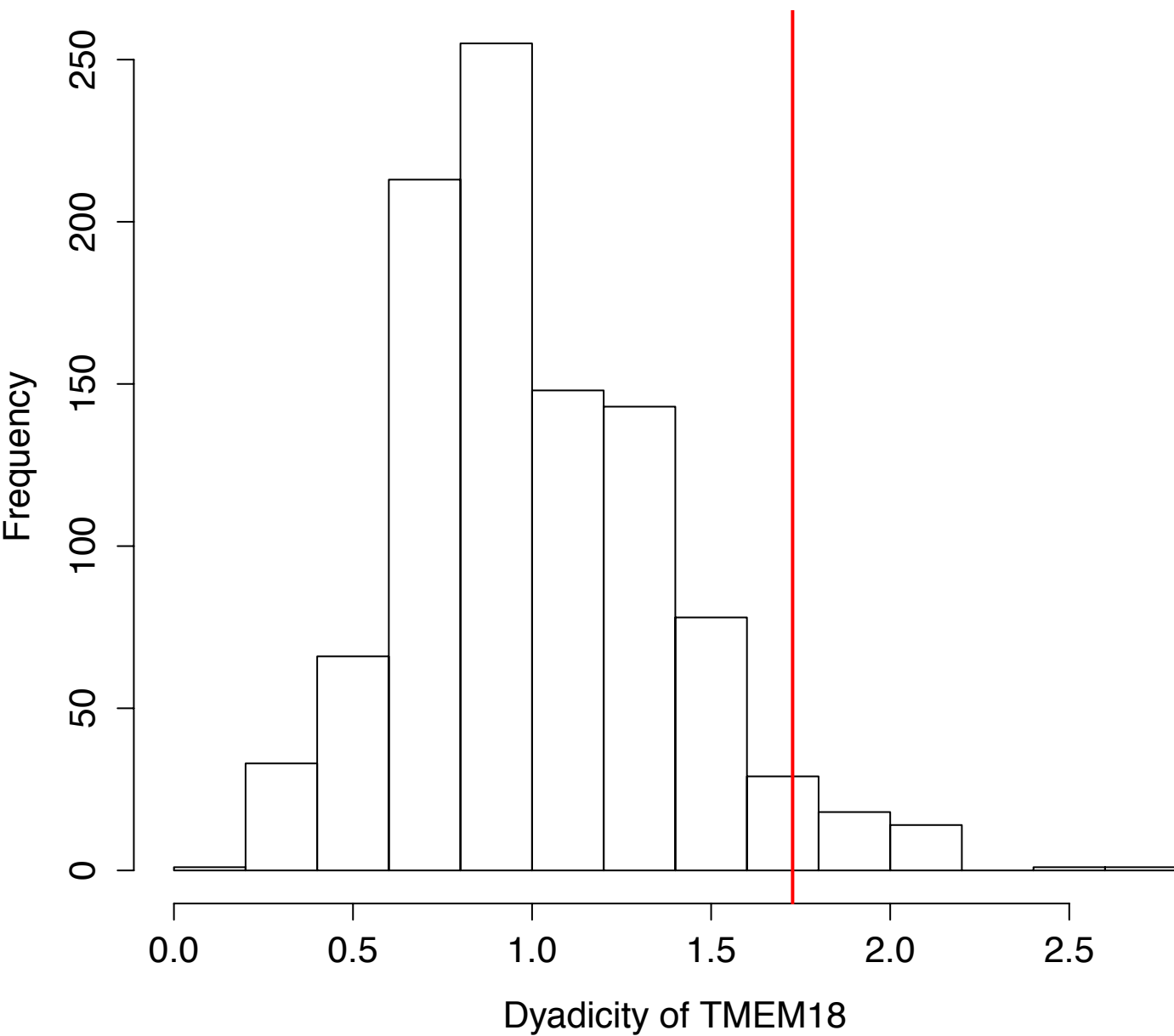

Supplement: Additional file 7: Figure S4. — Null distribution of dyadicity values of TMEM18 from 1000 permuted networks. Null distribution of dyadicity values of TMEM18 from 1000 permuted networks. The red line indicates the observed heterophilicity value of TMEM18 within the real data network. (PDF 12 kb) [file 13040_2015_77_MOESM7_ESM.pdf]

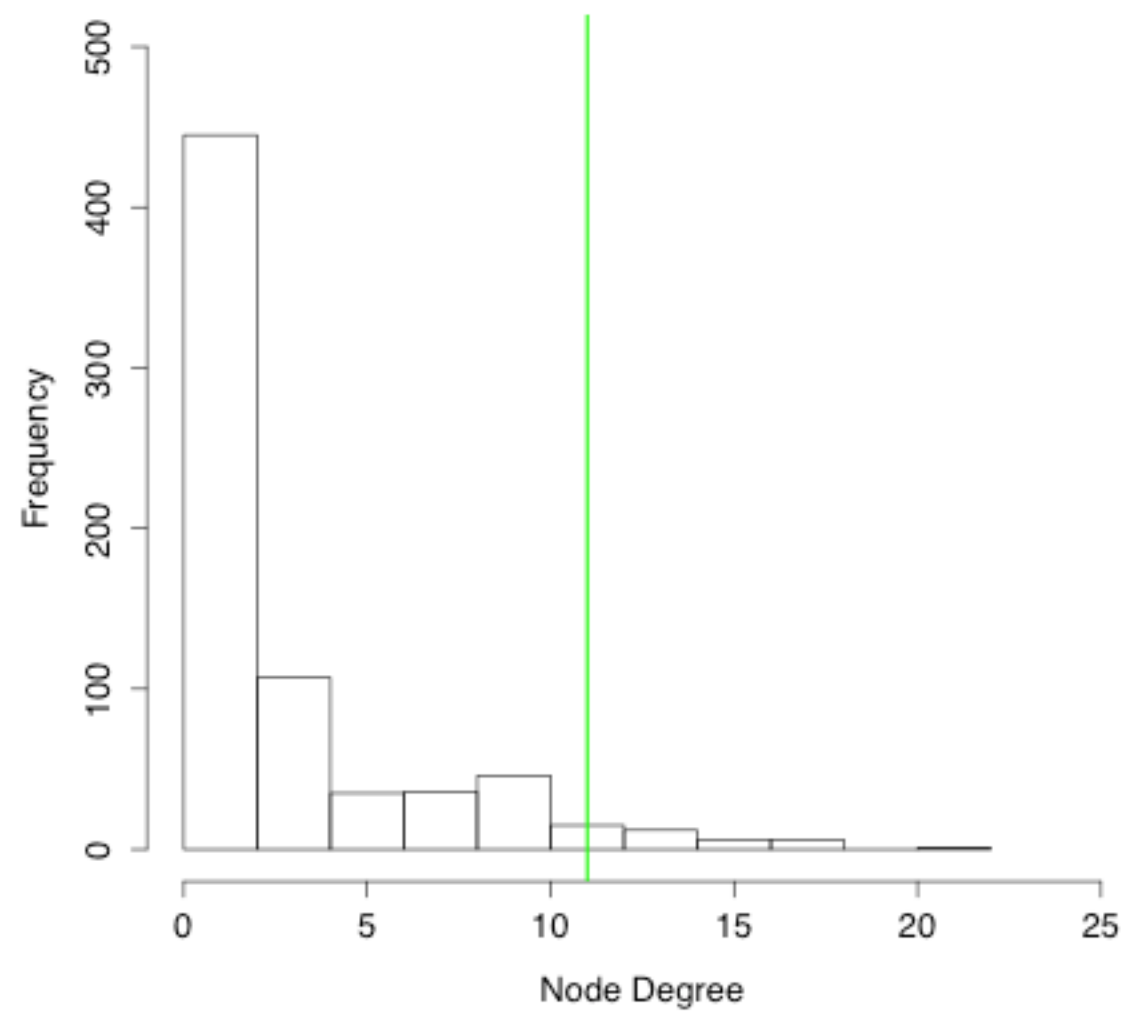

Supplement: Additional file 9: Figure S5. — Frequency distribution of node degree values within the SEN. Frequency distribution of node degree of 709 SNPs within the giant connected component of the SEN. The green line indicates the top 5 % of node degree values. (PDF 18 kb) [file 13040_2015_77_MOESM9_ESM.pdf]

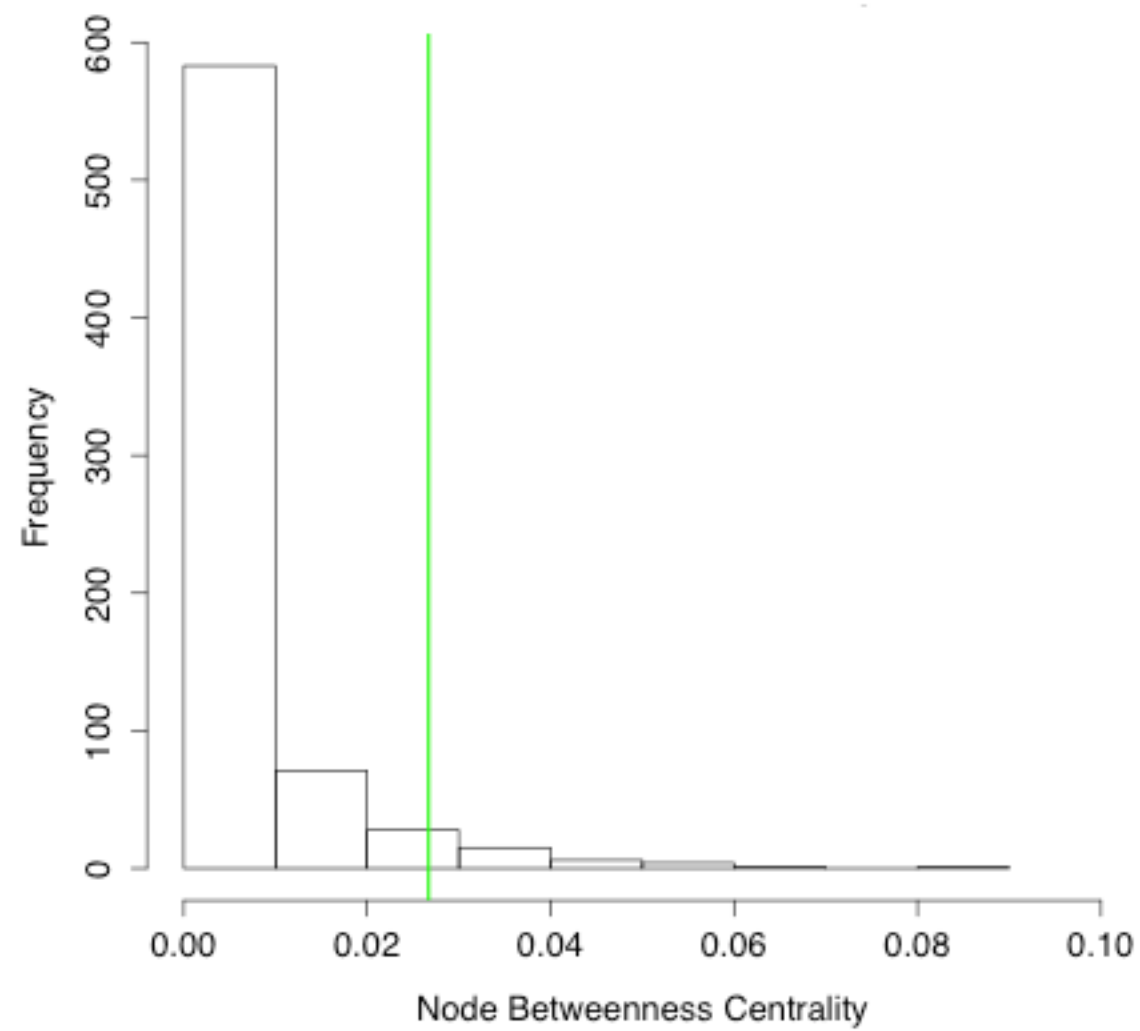

Supplement: Additional file 10: Figure S6. — Frequency distribution of node betweenness centrality values within the SEN. Frequency distribution of node betweenness centrality measures of 709 SNPs within the giant connected component of the SEN. The green line indicates the top 5 % of betweenness centrality values. (PDF 20 kb) [file 13040_2015_77_MOESM10_ESM.pdf]

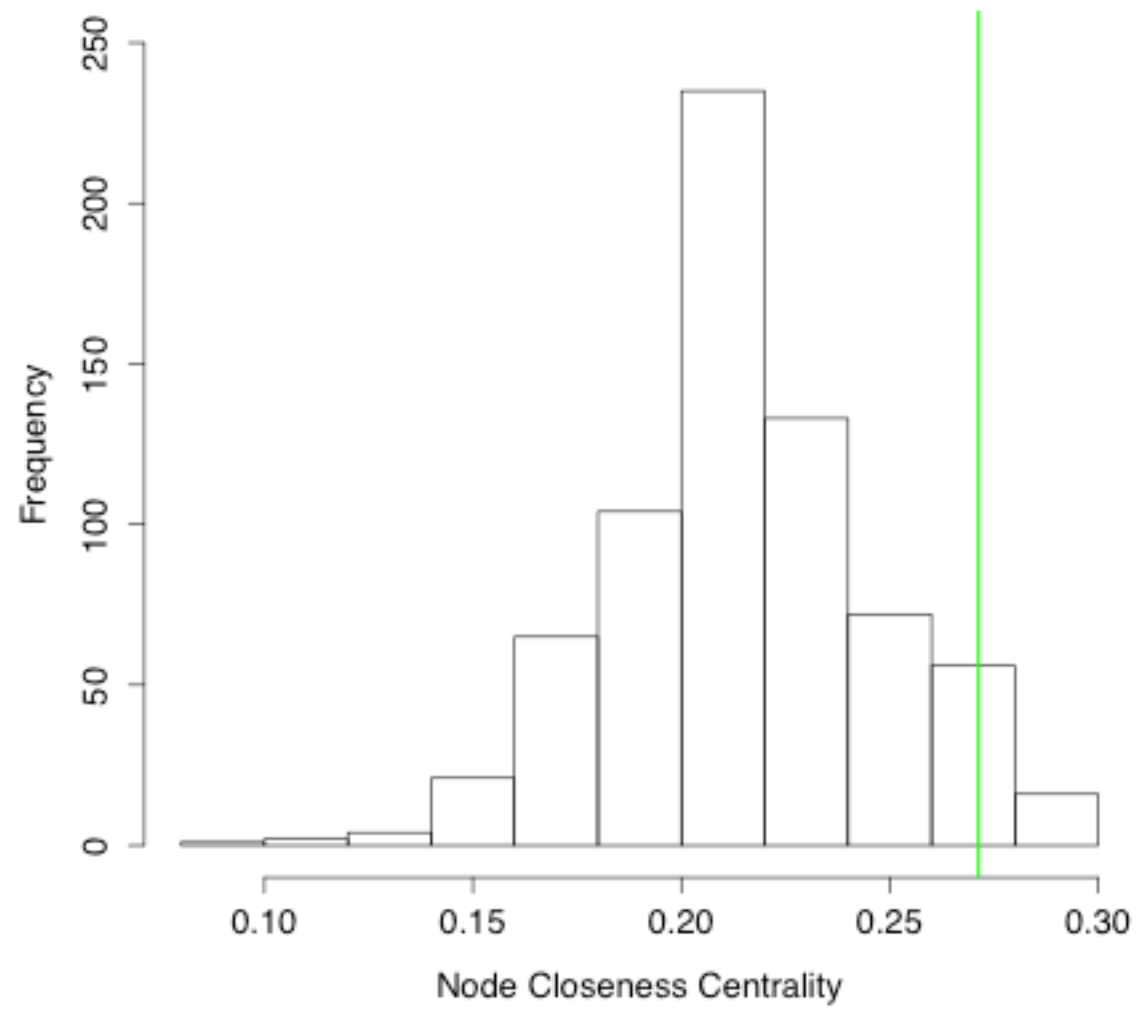

Supplement: Additional file 11: Figure S7. — Frequency distribution of node closeness centrality values within the SEN. Frequency distribution of node closeness centrality measures of 709 SNPs within the giant connected component of the SEN. The green line indicates the top 5 % of closeness centrality values. (PDF 21 kb) [file 13040_2015_77_MOESM11_ESM.pdf]
